# The oldest patient in literature of spontaneous true giant superficial temporal artery aneurysm

**DOI:** 10.1016/j.jtumed.2022.06.010

**Published:** 2022-07-09

**Authors:** Yasin Ozden, Osman E. Karpuzoglu, Umit Hardal, Tufan Sener

**Affiliations:** a Department of Cardiovascular Surgery, University of Health Sciences, Dr. Siyami Ersek Thoracic and Cardiovascular Surgery Training and Research Hospital, Istanbul, Turkey; b Department of Ear Nose Throat, Dr Hardal ENT Clinic, Private Practise, Istanbul, Turkey; c Department of Cardiovascular Surgery, University of Health Sciences, Haseki Training and Research Hospital, Istanbul, Turkey

**Keywords:** Pulsatil mass, Saccular aneurysm, Superficial temporal artery, Temporal region, True aneurysm

## Abstract

**Background:**

Superficial temporal artery (STA) aneurysms are very rare compared to vascular aneurysms of other regions. They are divided into two as true and pseudo. Pseudoaneurysm were much more common and often depend on an etiological factor but spontaneous true aneurysms are extremely uncommon and the etiologic causes are not clear yet.

**Case presentation:**

We present a 91-year-old female patient who consulted to us with swelling in front of the ear; there was no history of previous surgery or any trauma. The patient had a pulsatile mass in the preauricular region, which started 4 years ago and growed faster for the last 2 months.

**Conclusion:**

There was a mass consistent with a saccular type aneurysm whose continuity was observed with the temporal artery in imaging studies. The mass was excised under general anesthesia. The patient whose pathological examination was a true STA aneurysm was discussed in the light of the literature.

Dear Editor, aneurysms of the superficial temporal artery (STA) are a scarce kind of aneurysms. The majority of this artery's aneurysms are pseudoaneurysms of posttraumatic origin, mostly due to blunt traumas, surgery, or even hair transplantation. Spontaneous true aneurysms are extremely uncommon,^1^ and the etiologic causes are not clear yet. Approximately 386 cases have been reported since 1644 and about 5% of the cases were true aneurysms.^2^ Very few of these cases were larger than 25 mm.

We present the case of a true STA aneurysm of 35 mm in diameter.

Our case; caucasian female, 91 years old, presented with a pulsatile mass over the right preauricular area, which was gradually getting more prominent for the last four years but expanded faster over the previous two months, and the pain was added. There wasn't any history of trauma or surgical intervention.

Examination revealed a 4 × 4 cm, partially mobile mass with pulsation superior to the right parotid gland. The skin over the mass was intact; no other aneurysms in any other region of the patient's body was found. There was no sign of inflammatory diseases, including several kinds of vasculitis.

Doppler Ultrasound examination showed a saccular aneurysm with dense echogenic thrombus material inside next to the parotid gland. Continuity with temporal artery and blood flow through the mass was seen Figure 1.

**Figure 1:**
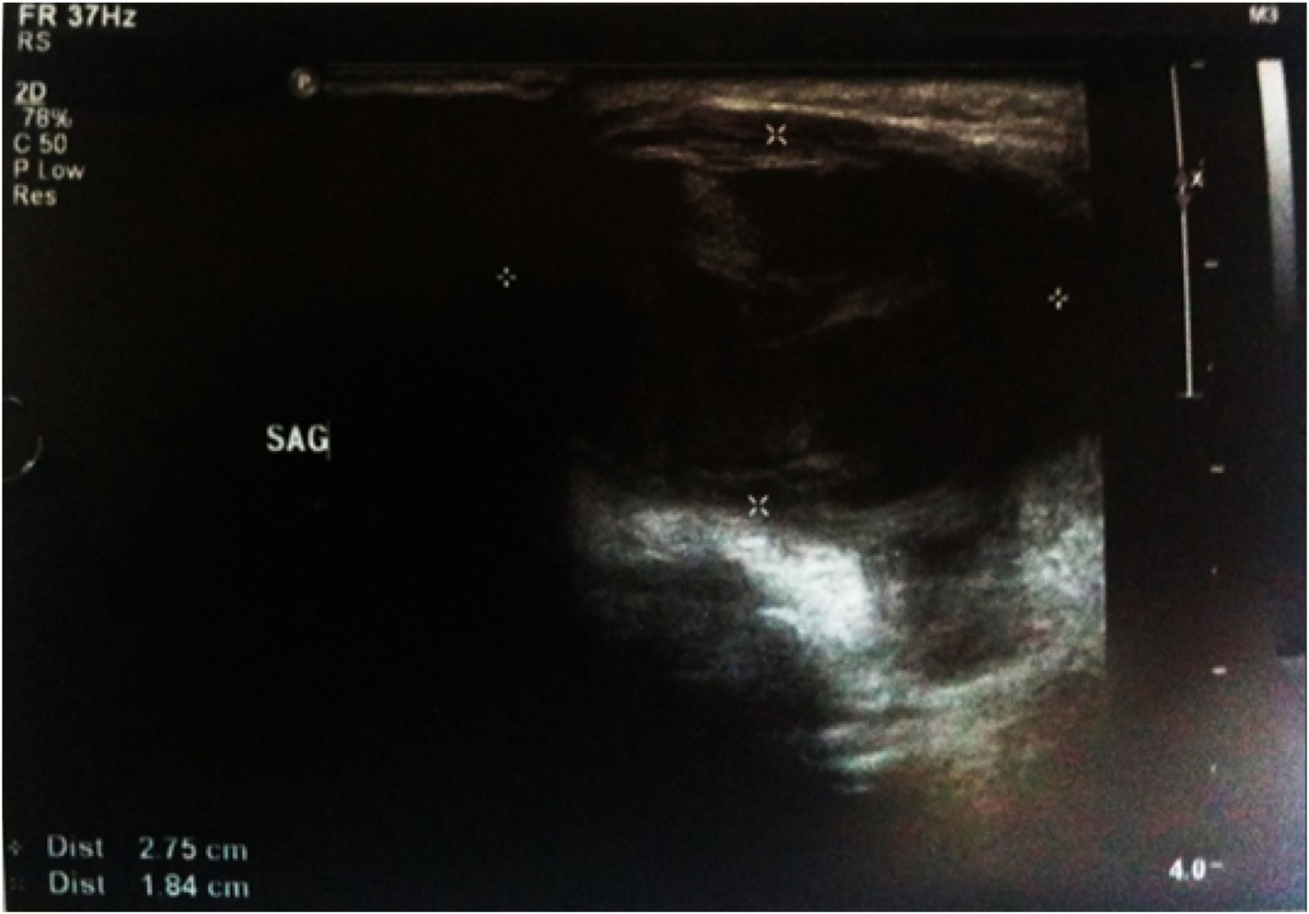
Doppler USG view of the mass.

Surgical excision of the aneurysm after dissection and ligation of the STA proximal and distal through a preauricular skin incision under general anesthesia was done Figure 2. Pathological examination revealed a 35 × 30 × 25 mm mass consisting of all layers (intima, media, and adventitia) of the artery with atheromatic deposits at media and intimal layers Figure 3. No relapse was seen through the follow up for 18 months.

**Figure 2:**
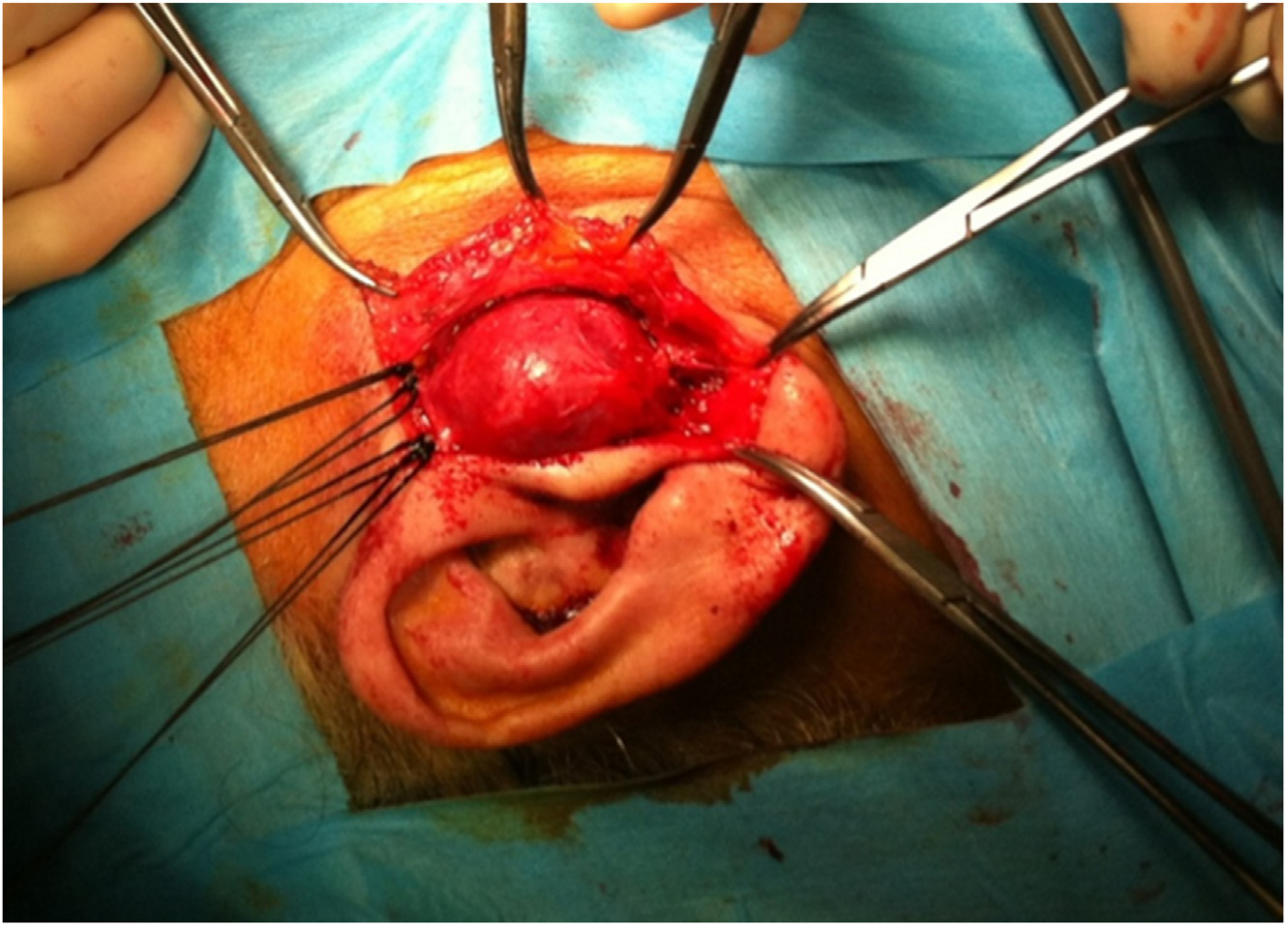
The view of the mass during the operation.

**Figure 3:**
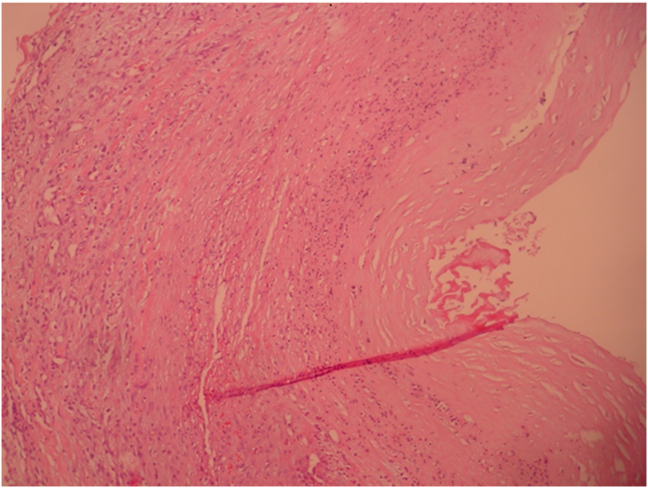
The view of the mass in 10×40 hemotocsilen eosin staining; all vessel layers are present.

Aneurysms associated with STA are only less than 1% of all arterial aneurysms reported up to date.^3^ Almost all of the reports were about pseudoaneurysms. Superficial location over the temporal bone and muscular tissue weakness around provides loose support to STA, making it vulnerable against minor and major traumas like high-velocity particles, including gunshot or other war-related wounds, blunt traumas, vehicle accidents, and sports injuries. Generally, 2–6 weeks after the incident, aneurysms begin to appear.^4^

In the review made by Kawai et al., In 2014, only 28 true spontaneous aneurysms were found and the oldest patient was 85 years old [10]. Considering that a few cases are added to this number of cases nowadays, this is around 30–35 cases and it is quite low. However no case as old as our case has been reported so far.

Spontaneous aneurysms of STA mean that there shouldn't be any kind of trauma in the patient's history. These true aneurysms were usually related to atherosclerosis, congenital weakness of vascular layers, or chronic arteritis.^5^^,^^6^

There weren't any history of trauma, metabolic disorder, infection, or systemic illness in our patient.

Most of the patients were presented with a pulsatile mass over the temporal area at the physical examination; some might be non-pulsatile due to thrombosis.^7^ Differential diagnosis includes aneurysms of arteria meningia media, arteriovenous fistulas or malformations (AVF/AVM), neuromas of the facial nerve, abscess, mass related with parotid gland, inclusion cysts or lipomas.^8^

Doppler USG is usually enough for diagnosis if there is a single and small aneurysm. If there are neurological symptoms, then detailed radiological evaluation of intracranial structures with angiography, computed tomography, or magnetic resonance imaging is indicated. The risk of stroke and neurological injury due to angiography must be kept in mind. These invasive techniques must be preserved for chosen patients with the probability of AVF or AVMs. Doppler USG may reveal native arteries with a fusiform dilatation, the presence of turbulent intraluminal blood flow, and partial or total thrombosis.^9^ We found Doppler USG sufficient for diagnosis because it showed the vascular structures well defined with the aneurysm, and the patient had no neurological or systemic symptoms.

Treatment options include interventional embolization or surgery. Expanding mass, pain, cosmetic issues, and degeneration of the skin or bone around the aneurysm indicate surgical excision.^10^ Our case was presented with an expanding pulsatile mass and severe pain. The considerable size and proximity to the skin of the aneurysm make it unsuitable for embolization. General anesthesia was preferred because of the size of the mass and close localization to the facial nerve and a successful surgery was performed.

True aneurysms of the STA are rare disorders, but they must be kept in mind when a pulsatile or non-pulsatile mass is seen in the temporal region. Although it can be seen in all age groups, there are no elderly patients as much as our case in the literature.

## Source of funding

This research did not receive any specific grant from funding agencies in the public, commercial, or not for- profit sectors.

## Conflict of interest

No conflict of interest.

## Ethical approval

This is a retrospective case report and required no ethical approval. An informed consent to publish the data and pictures was obtained from the patient.

## Authors contributions

YO: Drafting the work or revising it critically for important intellectual content. UH, TS: Agreement to be accountable for all aspects of the work in ensuring that questions related to the accuracy or integrity of any part of the work are appropriately investigated and resolved. OEK: Final approval of the version to be published. All authors have critically reviewed and approved the final draft and are responsible for the content and similarity index of the manuscript.
